# Data on mixed trophies biofilm for continuous cyclohexane oxidation to cyclohexanol using *Synechocystis* sp. PCC 6803

**DOI:** 10.1016/j.dib.2019.104059

**Published:** 2019-05-25

**Authors:** Ingeborg Heuschkel, Anna Hoschek, Andreas Schmid, Bruno Bühler, Rohan Karande, Katja Bühler

**Affiliations:** Department of Solar Materials Helmholtz-Centre for Environmental Research, UFZ Permoserstrasse 15, 04318 Leipzig, Germany

**Keywords:** Biofilm monitoring, Biofilm cultivation, Confocal laser scanning microscopy, Cyclohexane conversion

## Abstract

Photosynthetic microorganisms offer promising perspectives for the sustainable production of value-added compounds. Nevertheless, the cultivation of phototrophic organisms to high cell densities (HCDs) is hampered by limited reactor concepts. Co-cultivation of the photoautotrophic *Synechocystis* sp. PCC 6803 and the chemoheterotrophic *P. taiwanensis* VLB 120 enabled HCDs up to 51.8 g_CDW_ L^−1^. Respective biofilms have been grown as a biofilm in capillary flow-reactors, and oxygen evolution, total biomass, as well as the ratio of the two strains, have been followed under various cultivation conditions. Furthermore, biofilm formation on a microscopic level was analyzed via confocal laser scanning microscopy using a custom made flow-cell setup. The concept of mixed trophies co-cultivation was coupled to biotransformation, namely the oxyfunctionalization of cyclohexane to cyclohexanol. For benchmarking, the performance of the phototrophic reaction was compared to the chemical process, and to a biotechnological approach using a heterotrophic organism only. The data presented refer to our research paper “Mixed-species biofilms for high-cell-density application of Synechocystis sp. PCC 6803 in capillary reactors for continuous cyclohexane oxidation to cyclohexanol” Hoschek et al., 2019.

**Table:** Specifications table

Subject area	Biotechnology
More specific subject area	Phototrophic mixed trophies biofilm cultivation and biotransformation
Type of data	Table, text file, graph
How data was acquired	Gas chromatography, dissolved oxygen measurement via oxygen microsensor, surface calculation of 3 D reconstructed images acquired by confocal laser scanning microscopy
Data format	*analyzed*
Experimental factors	The biofilm was cultivated in a custom made flow-cell developed for *in-vivo* confocal microscopy. For the dissolved oxygen determination in the liquid phase of the capillary reactors, microsensors were used. Whereas, oxygen in the gas phase was quantified via gas chromatography. For biomass analysis, the total biomass was mechanically removed from the capillary, re-suspended, and taken directly for coulter counter measurements and cell dry weight determination. To follow cyclohexane conversion, the substrate and the product were extracted from the aqueous phase with diethyl ether and quantified directly by gas chromatography.
Experimental features	The biofilm was either cultivated in the custom made flow-cell or in the capillary flow-reactor
Data source location	*Leipzig, Germany*
Data accessibility	All data are available in this document and in the Mendeley data sets (https://doi.org/10.17632/vgx2hgxsc4.1).
Related research article	“Mixed-species biofilms for high-cell-density application of Synechocystis sp. PCC 6803 in capillary reactors for continuous cyclohexane oxidation to cyclohexanol”, Bioresource Technology, 282, (2019), 171–178 [1].

**Table:** 

**Value of the data** • The biofilm growth and development in a mixed-trophies format was investigated to gain insight on the role of individual species in biofilm formation. • The recorded data of oxygen produced per biomass and the ratio between different species are valuable to understand and optimize high cell density culture of phototrophic organisms. • The applicability of the bioreactor concept was demonstrated by continuous biotransformation of volatile and toxic substrates. • For benchmarking, the performance of the mixed-trophies biofilm was compared to the chemical process, and to a biotechnological approach using a heterotrophic organism only

## 1 Data

**Fig. 1:**
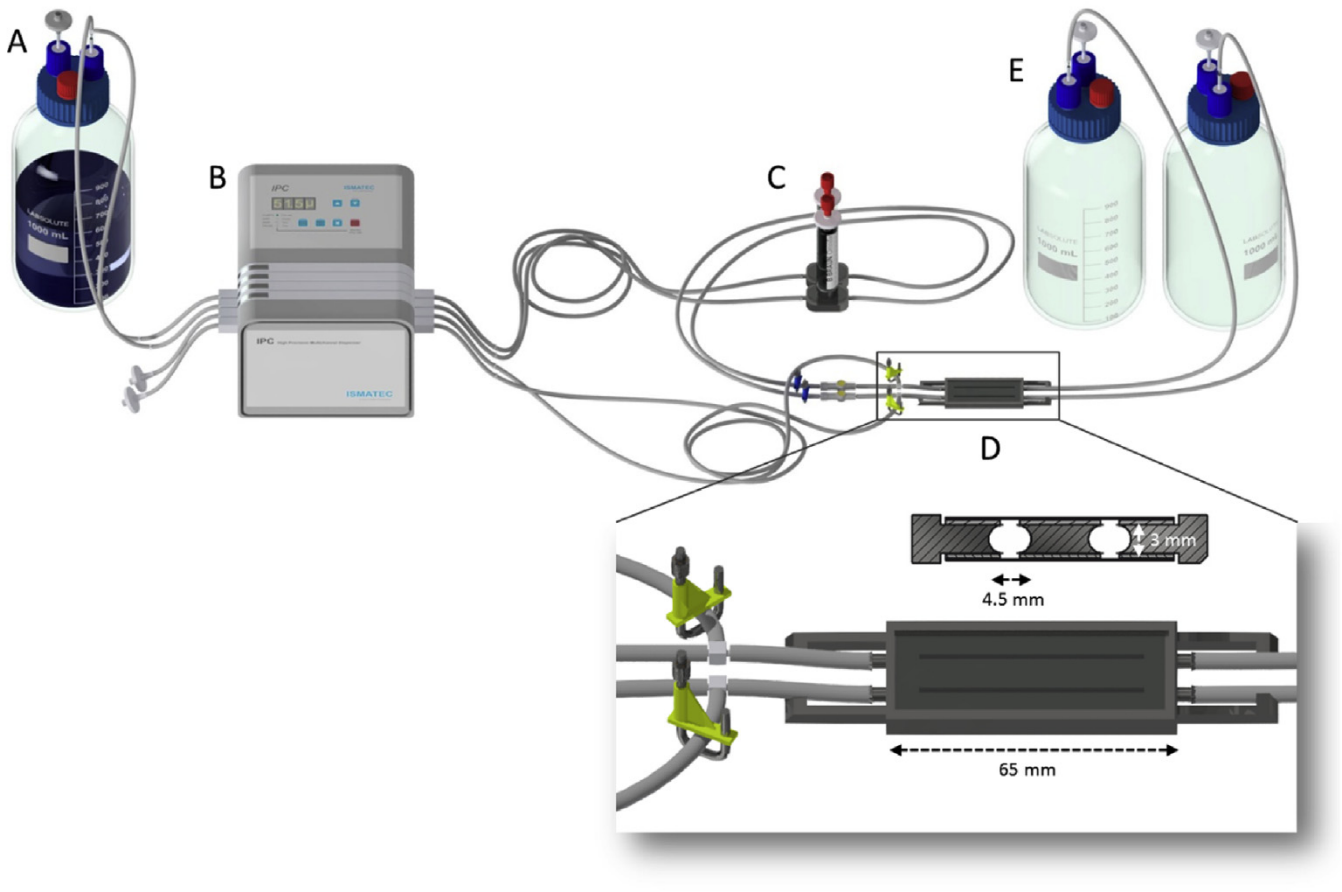
**Flow cell setup for biofilm analysis using CLSM.** Medium reservoir (A), a multichannel peristaltic pump (B, IPC 4 peristaltic pump, Ismatec), bubble trap (C), flow-cell (D), and waste bottles (E) with the respective tubing. An enlarged view of the flow cell (D), as well as the cross-section, is shown. The body of the flow cell is made of stainless steel, whereas the top, and bottom parts are made of glass to enable microscopic images.

This dataset contains information on strain development, biofilm cultivation devices, and imaging techniques, as well as analysis tools for characterizing productive biofilms converting cyclohexane to cyclohexanol. Bacterial strains and plasmids used for biocatalyst development are listed in Table 1, and their genetic features are briefly described. The schematic representation of the cultivation system developed for biofilm imaging using a confocal laser scanning microscope (CLSM) is given in Fig. 1. The central cultivation device is a flow cell made of stainless steel with the dimensions 65 mm × 4.5 mm fitting beneath the microscope. The respective volumina of the biological specimen recorded using CLSM were calculated after 3D reconstruction from the acquired images using Imaris 8.2.0 [1] and are presented in Table 2.

**Table 1:** Strains, plasmids, and primers used in this study.

Strain	Description	Reference
*E. coli* DH5α	F^−^ Φ80lacZΔM15 Δ(lacZYA-argF) U169 recA1 endA1 hsdR17 (rK^–^, mK^+^) phoA supE 44 λB^–^ thi^−1^ gyrA96 relA1	[2]
*Synechocystis* sp. PCC 6803	Geographical origin: California, USA; Received from Pasteur Culture Collection of Cyanobacteria (PCC, Paris, France)	[3]
*Pseudomonas taiwanensis* VLB120	Wild-type *Pseudomonas*; styrene prototroph	[4]
*Pseudomonas taiwanensis* VLB120_egfp	*P. taiwanensis* VLB120 harboring a chromosomally integrated *egfp* (enhanced green fluorescent protein) gene	This study
Plasmid	Description	Reference
pAH032	pPMQAK1 based, RSF ori, Kanamycin resistance, empty P_*trc1O*_ expression system	[5]
pAH050	Based on pAH032; CYP, FnR, and Fn genes under control of P_*trc1O*_ promoter system, RBS* optimized for *Synechocystis* sp. PCC 6803 in front of CYP gene	[6]

**Table 2:** Volumina of *P. taiwanensis* VLB 120_egfp and *Synechocystis* sp. PCC 6803 (pAH050) has been calculated from the CLSM images presented in [1].

	*P. taiwanensis* VLB120_egfp [μm^3^]	*Synechocystis* sp*.* PCC 6803 (pAH050) [μm^3^]	Volume ratio (*Ps*/*Syn)*
Inoculation	5.9 × 10^3^	1.6 × 10^4^	0.37
1 day after medium flow	1.5 × 10^4^	4.4 × 10^4^	0.357
3 days	1.1 × 10^4^	5.4 × 10^4^	0.217
11 days	2.4 × 10^3^	1.0 × 10^5^	0.0239
25 days	3.1 × 10^3^	1.1 × 10^5^	0.0284

**Fig. 2:**
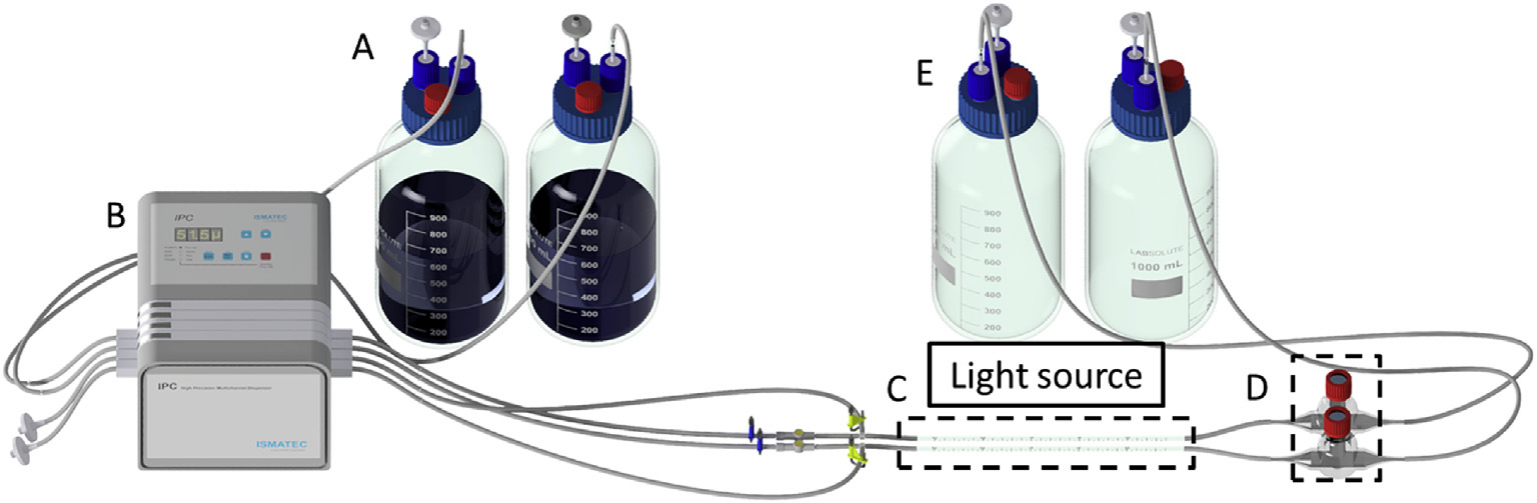
**Set-up of the biofilm capillary reactor system.** The figure shows the medium reservoir (A), a multichannel peristaltic pump (B), peristaltic pump from Ismatec, capillary reactors, dimensions: 20 cm × 0.3 cm (C) with the light source on the top, (D) bubble traps for gas phase sampling and (E) waste bottles.

**Fig. 3:**
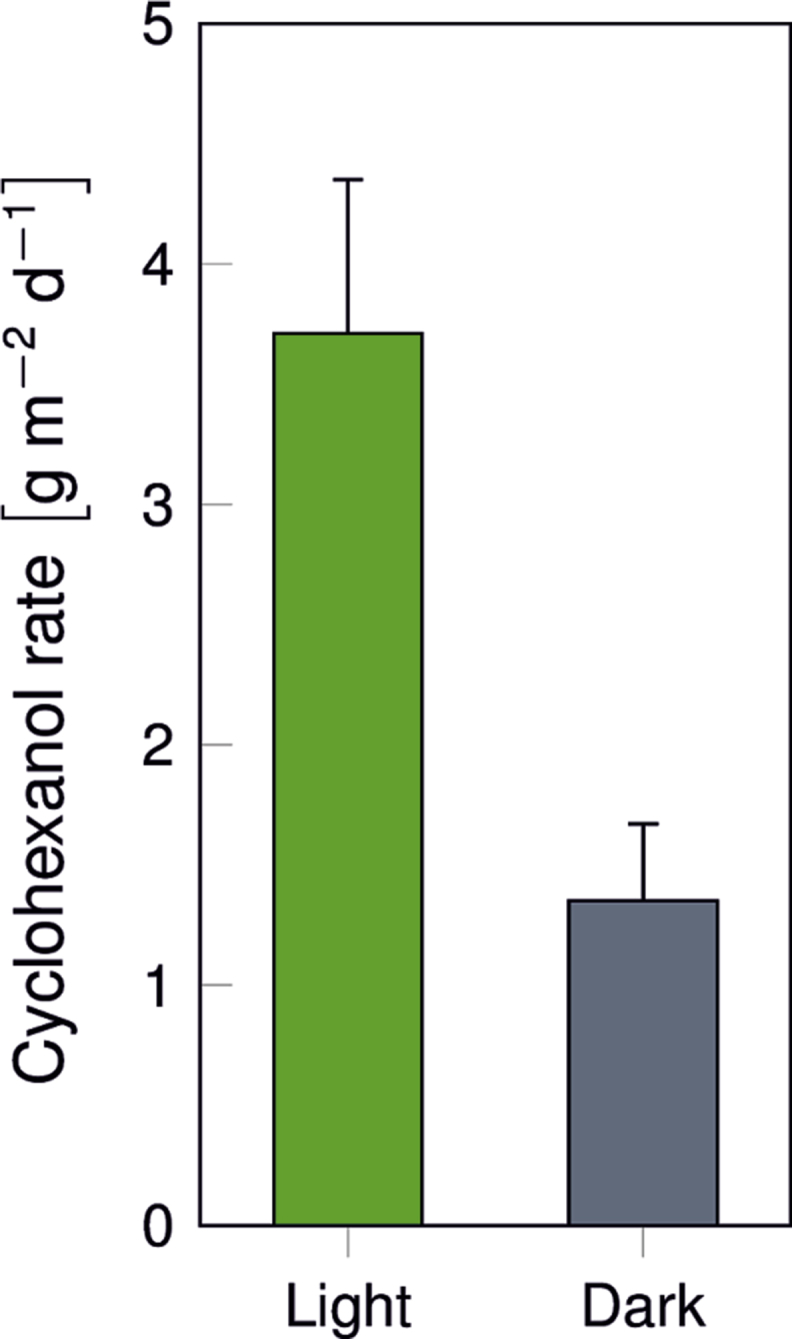
**The average cyclohexanol production rate in g_CHXOL_ m^−2^ d^−1^ utilizing *Synechocystis* sp. PCC 6803 (pAH050) *and P. taiwanensis* VLB 120 (pAH050) as a dual-species mixed-trophies biofilm under light and dark conditions.** Experiments were conducted at 50 μE m^−2^ s^−1^ providing organic carbon free YBG11 medium and air segments at a flow rate of 52 μL min^−1^. Green and grey bars represent product formation under light and dark conditions, respectively. CHXOL = cyclohexanol.

In Fig. 2, a schematic representation of the biofilm reactor system developed for the transformation of cyclohexane to cyclohexanol using mixed trophies biofilms comprising photoautotrophic and chemoheterotrophic organisms is shown. Biofilms are cultivated in capillaries with the dimensions 20 cm × 0.3 cm. Performance parameters like oxygen concentration, citrate consumption, and biofilm dry weight are summarized in Table 3, while average cyclohexanol production rates in light and dark conditions are given in Fig. 3. Different process concepts for cyclohexanol production are compared in Table 4.

**Table 3:** Biofilm cultivation parameters for single- and dual-species capillary reactors. i) and ii) depict monoseptic biofilm cultures of *Synechocystis* sp. PCC 6803 (pAH032) without (-Air) and with air segments (+Air), respectively. Dual species biofilm cultures of *Synechocystis* sp. PCC 6803 (pAH032) and *P. taiwanensis* VLB120 (pAH032) were inoculated at a ratio of 1:1 and operated iii) without and iv) with air segments. v) and vi) correspond to iii) and iv) with citrate in the aqueous medium feed. The aqueous medium was fed at a rate of 52 μL min^−1^. For segmented flow, a gaseous air phase was additionally fed at the same rate.

Experimental setup		O_2_ in aq. Phase^a^ (μM)	Citrate Consumption (g L^−1^)	Biofilm dry weight^b^(g L^−1^)		
				Ps	Syn	total
i	- Air	746	–	–	1.5	1.5
ii	+ Air	284	–	–	13.7	13.7
iii	- Air	923	–	0.1	5.8	5.9
iv	+ Air	287	–	0.2	31.4	31.6
v	- Air	∼0	0.27	7.2	40.6	47.8
vi	+ Air	194	0.39	1.5	17.3	18.8

a Solubility of O_2_ at 26 °C and a salinity of 3.5 g kg^−1^: ∼250 μM (21% O_2_) and ∼1190 μM (100% O_2_); based on the respective partitioning, aqueous phase O_2_ concentrations given for experiments performed with air segments are calculated from O_2_ concentrations measured in the gas phase.

b The biofilm dry weight is calculated based on 1.2 mL tube volume. *Synechocystis* sp. PCC 6803 (pAH032) and *P. taiwanensis* VLB120 (pAH032) specific biofilm dry weights are calculated based on cell numbers and cell volumes and the respective total biofilm dry weight, assuming that both strains constitute equal biovolume to biofilm dry weight ratio.

**Table 4:** Comparison of key parameters of different reaction concepts for cyclohexanol production.

Parameters	Conventional process^a^	Heterotrophic bioprocess^b^	Phototrophic bioprocess^c^
Reaction temperature (K)	413–453	303	295
Pressure (atm)	7–20	1	1
Residence time (min)	7–20	5–16	13
Cyclohexane conversion (%)	ca. 6	NA	98.9
Combinatorial selectivity (%)	80–90	NA	100
Space-time-yield (g L^−1^ h^−1^)	ca. 25	ca. 0.4^d^	ca. 0.2^e^

NA: not available.

Combinatorial selectivity refers to the formation of cyclohexanol and cyclohexanone.

a Data from reference [8].

b Data from reference [9].

c This study.

d In complex medium (LB media).

e In minimal medium (YBG11 media).

## 2 Experimental design, materials, and methods

Bacterial strains and plasmids used in this study are listed in Table 1. Additionally, the composition of YBG11 media used in this study is given.

**Composition of YBG11 (50 mM NaHCO_3_, without citrate):** 1.49 g L^−1^ NaNO_3_, 0.074 g L^−1^ MgSO_4_ 7H_2_O, 0.0305 g L^−1^ K_2_HPO_4_, 10 mL L^−1^ YBG11 trace elements (100x), 0.019 g L^−1^ Na_2_CO_3_, 50 mM HEPES (pH 7.2); YBG11 trace elements (100x): 3.6 g L^−1^ CaCl_2_ 2H_2_O, 0.28 g L^−1^ boric acid, 0.11 g L^−1^ MnCl_2_ 4H_2_O, 0.02 g L^−1^ ZnSO_4_ 7H_2_O, 0.039 g L^−1^ Na_2_MoO_4_ 2H_2_O, 0.007 g L^−1^ CuSO_4_ 5H_2_O, 0.005 g L^−1^ Co(NO_3_)_2_ 6H_2_O, 0.162 g L^−1^ FeCl_3_ 6H_2_O, 0.6 g L^−1^ Na_2_EDTA 2H_2_O, 4.2 g L^−1^ NaHCO_3_ [7].

### 2.1 Monitoring biofilm growth in a flow-cell by confocal laser scanning microscopy

The development of a mixed trophies biofilm consisting of *P. taiwanensis* VLB 120_egfp and *Synechocystis* sp. PCC 6803 (pAH050) was analyzed by confocal laser scanning microscopy (CLSM). The schematic representation of the experimental set-up is given in Fig. 1. The respective volumina of the biological specimen were calculated after 3D reconstruction from the acquired images using Imaris 8.2.0 [1] and are presented in Table 2. The eGFP signal of *Pseudomonas* sp. as well as the autofluorescence of *Synechocystis* sp. was recorded individually so that the volume could be calculated for each channel individually. The total volume of the biofilm equals the sum of the volume occupied by each species. *Ps* = *P. taiwanensis* VLB120_egfp, *Syn* = *Synechocystis* sp. PCC 6803 (pAH050). For the volume ratio of Ps/Syn, the calculated volume of *Pseudomonas* sp. was divided by the volume of *Synechocystis* sp. PCC 6803.

### 2.2 Biofilm cultivation in capillary reactors

Biofilms were cultivated as mixed and single species biofilms of *Synechocystis* sp. PCC 6803 and *P. taiwanensis* VLB120 both containing the plasmid pAH032. The schematic representation of the experimental set-up is given in Fig. 2.

The amount of oxygen produced as well as the biofilm dry weight and composition regarding bacterial species was determined for each cultivation condition (Table 3). For further cultivation details, please refer to [1].

### 2.3 Biotransformation of cyclohexane to cyclohexanol in capillary reactors

After 36 days of cultivation, the biotransformation was initiated for a mixed species biofilm of *Synechocystis* sp. PCC 6803 (pAH050) and *P. taiwanensis* VLB 120 (pAH050) by the addition of cyclohexane. The biotransformation substrate cyclohexane was supplied via saturation of the medium and air phase by a silicon membrane, before the reactor inlet. The productivity of 3.76 g_CHXOH_ m^−2^ day^−1^ was reached after 1 day of adaptation and was stable for 30 days. After 31 days, the setup was actively terminated [1]. The light was turned off during day 8 and 10 so that *Synechocystis* sp. PCC 6803 (pAH050) was no longer able to perform photosynthesis, and this resulted in to decrease of the productivity 1.0–1.3 g_CHXOH_ m^−2^day^−1^ [1]. The average volumetric productivities during the light and dark conditions are 3.71 g_CHXOH_ m^−2^ day^−1^ and 1.35 g_CHXOH_ m^−2^ day^−1^, respectively (Fig. 3).

### 2.4 Benchmarking

The here presented biotransformation using a mixed-trophies biofilm consisting of a photoautotrophic and a chemoheterotrophic strain has been compared to the conventional chemical process and to a biotechnological approach using a heterotrophic organism only (Table 4). Thereby, the advantages and disadvantages of the different concepts become obvious, and new engineering targets may be identified to develop an economic and sustainable process.
